# Characterization of *Campylobacter* spp. isolated from wild birds in the Antarctic and Sub-Antarctic

**DOI:** 10.1371/journal.pone.0206502

**Published:** 2018-11-09

**Authors:** Håkan Johansson, Patrik Ellström, Karin Artursson, Charlotte Berg, Jonas Bonnedahl, Ingrid Hansson, Jorge Hernandez, Juana Lopez-Martín, Gonzalo Medina-Vogel, Lucila Moreno, Björn Olsen, Eva Olsson Engvall, Hanna Skarin, Karin Troell, Jonas Waldenström, Joakim Ågren, Daniel González-Acuña

**Affiliations:** 1 Centre for Ecology and Evolution in Microbial Model Systems, Linnaeus University, Kalmar, Sweden; 2 Zoonosis Science Center, Department of Medical Biochemistry and Microbiology, Uppsala University, Uppsala, Sweden; 3 Department of Medical Sciences, Uppsala University, Uppsala, Sweden; 4 National Veterinary Institute, Uppsala, Sweden; 5 Department of Animal Environment and Health, Swedish University of Agricultural Sciences, Skara, Sweden; 6 Department of Infectious Diseases, Kalmar County Hospital, Kalmar, Sweden; 7 Department of Biomedical Sciences and Veterinary Public Health, Swedish University of Agricultural Sciences, Uppsala, Sweden; 8 Laboratory of Microbiology, Kalmar County Hospital, Kalmar, Sweden; 9 Departamento de Patología y Medicina Preventiva, Facultad de Ciencias Veterinarias, Universidad de Concepción, Chillán, Chile; 10 Centro de Investigación para la Sustentabilidad, Universidad Andrés Bello, Santiago, Chile; 11 Facultad de Ciencias Naturales y Oceanográficas, Universidad de Concepción, Concepción, Chile; 12 Facultad de Ciencias Veterinarias, Universidad de Concepción, Chillán, Chile; Massey University, NEW ZEALAND

## Abstract

A lack of knowledge of naturally occurring pathogens is limiting our ability to use the Antarctic to study the impact human-mediated introduction of infectious microorganisms have on this relatively uncontaminated environment. As no large-scale coordinated effort to remedy this lack of knowledge has taken place, we rely on smaller targeted efforts to both study present microorganisms and monitor the environment for introductions. In one such effort, we isolated *Campylobacter* species from fecal samples collected from wild birds in the Antarctic Peninsula and the sub-Antarctic island of South Georgia. Indeed, in South Georgia, we found *Campylobacter lari* and the closely related *Campylobacter peloridis*, but also distantly related human-associated multilocus sequence types of *Campylobacter jejuni*. In contrast, in the Antarctic Peninsula, we found *C. lari* and two closely related species, *Campylobacter subantarcticus* and *Campylobacter volucris*, but no signs of human introduction. In fact, our finding of human-associated sequence types of *C. jejuni* in South Georgia, but not in the Antarctic Peninsula, suggests that efforts to limit the spread of infectious microorganisms to the Antarctic have so far been successful in preventing the introduction of *C. jejuni*. However, we do not know how it came to South Georgia and whether the same mode of introduction could spread it from there to the Antarctic Peninsula.

## Introduction

The Antarctic is among the most isolated places on Earth. By virtue of inhabiting such a remote location, Antarctic animals were long thought to be protected from disease introduction from other regions. However, recent studies have reported the presence of human and animal pathogens previously believed to be absent from the region [1, 2], including *Salmonella enterica* serovar Enteriditis phage type 4 [3–5] and influenza A viruses [6]. In addition to finding pathogens with presumed non-Antarctic origin in Antarctic wildlife, it has been shown that penguins kept in captivity are susceptible to a range of infectious diseases not observed in the Antarctic (see [2], and references therein). Sustained transmission of some of these pathogens are unlikely, due to the absence of suitable vectors in the Antarctic. Others may only be limited by geographical barriers. The breakdown of such barriers due to human activity may therefore pose a threat to the Antarctic ecosystem.

There has been no causal evidence of human-mediated pathogen introduction to the Antarctic [7]. However, due to a lack of knowledge concerning naturally occurring pathogens in the region, it is difficult to determine whether a detected pathogen has been introduced by humans or not. Furthermore, any study of disease in the Antarctic faces several challenges, including the environment, which poses a major hurdle to longitudinal monitoring of individuals and populations, and limited access to sufficient laboratory infrastructure, which makes the study of fastidious microorganism difficult. Nevertheless, overcoming these obstacles and furthering our understanding of disease in the region is a priority for both conservation efforts and our ability to use the Antarctic to study human impact on a relatively uncontaminated environment [7–9].

In the present study, we focused on *Campylobacter*, a genus of bacteria that are often found in the gut microbiota of both wild and domestic animals, especially in avian species [10]. This genus includes *Campylobacter jejuni*, one of the leading causes of bacterial gastroenteritis in humans (*e.g.* [11–13]). At least five species of *Campylobacter* have been found in the Antarctic and the surrounding sub-Antarctic: *Campylobacter insulaenigrae* [14], *Campylobacter jejuni* [15, 16], *Campylobacter lari* [14, 17, 18], *Campylobacter subantarcticus* [19] and *Campylobacter volucris* [18]. In addition, at least one unidentified *C. lari*-like bacterium has been reported [20]. So far, three isolates of *C. jejuni* ST-45 from Macaroni penguins (*Eudyptes chrysolophus*) on Bird Island, South Georgia, constitutes the only detection plausibly associated with human activity [15, 16]. Therefore, the aim of our study was twofold: *i*) to look for potentially introduced *Campylobacter*, *i.e.* human-associated strains of primarily *C. jejuni*, and *ii*) to further increase our knowledge of *Campylobacter* spp. in the Antarctic and sub-Antarctic, particularly in light of recent characterizations of novel *C. lari*-like *Campylobacter* species [19, 21, 22].

## Materials and methods

### Ethics statement

Samples were collected in accordance with the Wildlife and Protected Areas (WPA) Ordinance enacted by the Government of South Georgia and the South Sandwich Islands, and the Protocol on Environmental Protection to the Antarctic Treaty. Permission to collect samples were granted by the Government of South Georgia and the South Sandwich Islands (WPA/2012/034), the Swedish Polar Research Secretariat (2012-169) and the Chilean Antarctic Institute (INACH 654/2014, 23/2015, 46/2016). Ethical consideration of sample methodology was approved by the Swedish animal ethics committee (Linköpings djurförsöksetiska nämnd, permits 112-11, 2-15).

### Sampling

Fieldwork was conducted during the austral summer in the Antarctic and Sub-Antarctic in four years. In November 2012, we sampled birds at three locations in South Georgia: Stromness (-54.16°, -36.71°), Grytviken (-54.27°, -36.51°) and Gold Harbor (-54.63°, -35.93°); and six locations in the Antarctic Peninsula: Danco Harbor (-64.73°, 62.59°), Deception Island (-62.98°, -60.65°), Orne Harbor (-64.62°, -62.53°), Paradise Harbor (-64.82°, -62.87°), Petermann Island (-65.17°, -64.14°) and Yankee Harbor (-62.53°, -59.77°). In January and February 2014, we sampled birds at five locations in the Antarctic Peninsula: Ardley Island (-62.21°, -58.93°), base Gabriel González Videla (-64.82°, -62.85°), Cape Legoupil (-63.32°, -57.90°), Kopaitik Island (-63.32°, -57.85°) and Neko Harbor (-64.84°, -62.53°). In January and February 2015, we sampled birds at three locations in the Antarctic Peninsula: Cape Shirreff (-62.46°, -60.79°), Kopaitik Island and Narebski Point (-62.24°, -58.78°). In January 2016, we sampled birds at four locations in the Antarctic Peninsula: Ardley Island, Cape Legoupil, Kopaitik Island and Rakusa Point (-62.16°, 58.46°).

In total, 2,278 samples were collected. Samples were predominantly collected from brush-tailed penguins (*Pygoscelis* spp.): Adélie penguins (*Pygoscelis adeliae*; n = 134), chinstrap penguins (*Pygoscelis antarctica*; n = 960) and gentoo penguins (*Pygoscelis papua*; n = 828). In addition, samples were collected from giant petrels (*Macronectes* spp.; n = 43), kelp gulls (*Larus dominicanus*; n = 151), king penguins (*Aptenodytes patagonicus*; n = 27), skuas (*Stercorarius* spp.; n = 46) and snowy sheathbills (*Chionis albus*; n = 89).

Sampling strategy is one factor that can affect prevalence estimates. Bearing this in mind, samples were obtained from birds captured with hand nets or from fresh feces directly from the nest when possible; when not, fecal samples were obtained from the spots on the ground where the birds had been seen standing still for a while, either alone or in single-species groups. In the latter case—which was particularly common for king penguins, kelp gulls, skuas and snowy sheathbills—care was taken to avoid droppings involving material from more than one bird. Consequently, the risk of one sample containing bacteria from several birds was limited, although occasional contamination cannot be ruled out.

Sampling methodology was similar in all years, and consisted of either fecal samples or cloacal swabs. Collected samples were kept in Amies charcoal medium (Copan Diagnostics, Inc. Murrieta, CA, USA) at +4°C. In 2012, the samples were kept refrigerated in Amies medium for about three weeks until they reached the Swedish National Veterinary Institute (SVA) where they were cultured immediately. In 2014, 2015 and 2016, the samples were kept in Amies charcoal medium for less than 24 h and then either cultured in a field-based laboratory (2015) or frozen to -70°C in lysogeny broth (LB) with 5% glycerol and transported in an unbroken freeze chain to Linnaeus University, Sweden (2014 and 2016). In the latter cases, the time from sampling to culturing was no longer than 3 months.

### Isolation and identification

All samples were enriched in Bolton broth (X135, Lab M, Lancashire, England; or CM0983, Oxoid, Basingstoke, England) supplemented with CVTN selective supplement (X132, Lab M) or modified Bolton broth selective supplement (SR0208, Oxoid,) and incubated at 37 ± 1°C for 48 ±4 h. Samples were plated on mCCDA (modified charcoal cefoperazone deoxycholate agar, SR0155, Oxoid) and incubated at 41.5 ± 0.5°C for 48 ± 4 h. Samples showing presumed *Campylobacter* growth were re-cultured on conventional blood agar and incubated at 41.5 ± 0.5°C for 48 ± 4 h. All incubations were performed in a microaerobic environment generated using CampyGen sachets (CN0025, Oxoid).

Isolates from 2012 were identified to species using phenotypic tests [23], PCR [24], and MALDI-TOF mass spectrometry [25]. Five of the isolates could not be unambiguously identified to species using MALDI-TOF. One of these isolates could not be analyzed further, but the remaining four were identified to species level by whole-genome sequencing and subsequent 16S rRNA gene analysis. Briefly, sequencing libraries were prepared using the Nextera XT kit (Illumina, San Diego, CA, USA) and 250 bp paired-end sequencing was performed on a MiSeq sequencer (Illumina). A partial (1,313 bp) 16S rRNA sequence that was shared between all *Campylobacter* spp. 16S rRNA gene sequences available in GenBank at the time (November, 2013) was identified and used as a reference sequence. For each isolate, the partial 16S rRNA gene sequence was determined by mapping the reads to the reference sequence using the crossmatch function of Consed [26]. The sequences were subsequently aligned with all *Campylobacter* spp. 16S rRNA gene sequences available in GenBank at the time (November, 2013), and a phylogenetic analysis was performed using MrBayes [27]. The four isolates (74507, 74514, 74521 and 74521) grouped with the *C. peloridis* reference sequence (GenBank accession number: AM922331) (see S1 Fig).

Isolates from 2014, 2015 and 2016 were identified to species following the *atpA* determination scheme developed by Miller *et al.* [28], supplemented with additional *atpA* reference sequences from *C. blaseri* 17S00004-5^T^ (GenBank accession number: MG958595), *C. ornithocola* WBE38^T^ (KX467979), *C. pinnipediorum* RM17260^T^ (CP012546), *C. hepaticus* HV10^T^ (LUKK01000000), *C. iguaniorum* 1485E^T^ (CP009043), *C. geochelonis* RC20^T^ (FIZP01000001), *C. corcagiensis* CIT 045^T^ (JFAP00000000). Briefly, the *atpA* gene was amplified and sequenced using a primer pair capable of targeting all known species of *Campylobacter* at the time of the schemes development (March, 2014). The sequences were subsequently aligned with the reference sequences using MAFFT v. v7.313 [29], and a phylogenetic analysis was performed using RAxML v. 8.2.9 [30]. All species formed monophyletic clades with the exception of *C. lari* which was paraphyletic with respect to *C. subantarcticus* (see S2 Fig). However, as there was strong support for the *C. subantarcticus* delimitation, samples falling within the larger *C. lari*-*C. subantarcticus* clade was treated as *C. subantarcticus* if they fell within the *C. subantarcticus*-clade and otherwise as *C. lari*.

All *C. jejuni* strains and a subset of the *C. lari* strains were typed using multilocus sequence typing (MLST) and the PubMLST databases (http://pubmlst.org/campylobacter/) as previously described [31–33].

## Results

We isolated *Campylobacter* in samples from the majority of the sampling locations and from almost all of the sampled species (Table 1, with detailed information in S1 Table). *Campylobacter* colonization was modest in penguins, nowhere exceeding 8.5%. The colonization was similarly modest in giant petrels (14.0%) and kelp gulls (13.9%), although locally it reached as high as 30.6% in kelp gulls. The colonization was markedly higher in skuas (50%) and sheathbills (48.3%) and in some locations reached 100% for these species. However, sample sizes were generally small for the non-penguin species.

**Table 1:** Occurrence of *Campylobacter* spp. in wild birds from South Georgia and the Antarctic Peninsula.

Year	Region	Location	Species	Positive (sampled)
2012	Antarctic Peninsula	Danco Harbor	Skua	0 (1)
			Snowy sheathbill	3 (3)
		Deception Island	Giant petrel	0 (1)
			Kelp gull	1 (63)*
		Orne Harbor	Kelp gull	0 (3)
			Snowy sheathbill	1 (4)
		Paradise Harbor	Snowy sheathbill	0 (2)
		Petermann Island	Kelp gull	0 (6)
			Snowy sheathbill	0 (1)
		Yankee Harbor	Skua	1 (5)
	South Georgia	Gold Harbor	Giant petrel	4 (22)
			Kelp gull	0 (1)
			King penguin	0 (27)
			Skua	4 (7)
			Snowy sheathbill	8 (12)
		Grytviken	Kelp gull	11 (36)
		Stromness	Giant petrel	3 (20)
			Kelp gull	3 (26)
			Skua	2 (9)
2014	Antarctic Peninsula	Ardley Island	Gentoo penguin	1 (160)
		Base Gabriel González Videla	Gentoo penguin	4 (92)
			Skua	8 (10)
			Snowy sheathbill	11 (17)
		Cape Legoupil	Gentoo penguin	6 (159)
			Skua	1 (1)
			Snowy sheathbill	13 (30)
		Kopaitik Island	Gentoo penguin	2 (342)
			Snowy sheathbill	7 (17)
		Neko Harbor	Gentoo penguin	0 (47)
			Kelp gull	6 (16)
			Skua	3 (6)
2015	Antarctic Peninsula	Cape Shirreff	Chinstrap penguin	2 (327)
		Kopaitik Island	Chinstrap penguin	31 (371)
		Narebski Point	Chinstrap penguin	2 (258)
2016	Antarctic Peninsula	Ardley Island	Adelie penguin	0 (31)
			Chinstrap penguin	0 (4)
			Gentoo penguin	0 (15)
			Skua	4 (7)
		Cape Legoupil	Adelie penguin	0 (1)
			Gentoo penguin	0 (13)
		Kopaitik Island	Adelie penguin	0 (87)
			Snowy sheathbill	0 (3)
		Rakusa Point	Adelie penguin	0 (15)

*The positive sample was identified as *C. lari*-like by MALDI-TOF, but could not be analyzed further.

Isolates recovered from the Antarctic Peninsula were identified as *C. lari* (75 isolates) or one of two closely related species: *C. subantarcticus* (25 isolates) and *C. volucris* (3 isolates). In addition, three isolates were identified as *C. lari*-like. *C. lari* was found in chinstrap and gentoo penguins, as well as kelp gulls, skuas and snowy sheathbills, whereas *C. subantarcticus* was only found in chinstrap penguins and a snowy sheathbill and *C. volucris* only in gentoo penguins (Table 2, with detailed information in S1 Table).

**Table 2:** Number of samples positive for each of the five species of *Campylobacter*. Numbers indicate samples for which species were determined by *atpA* sequencing; numbers in parentheses indicate additional samples for which species were determined by phenotypic tests, PCR and MALDI-TOF, but not by *atpA* sequencing. In the latter case, the methods used do not distinguish between *C. lari* and *C. subantarcticus* or *C. volucris*; these samples should therefore be considered positive for *C. lari*-like bacteria.

Region	Species	*C. jejuni*	*C. lari*	*C. peloridis*	*C. subantarcticus*	*C. volucris*
Antarctic Peninsula	Adelie penguin	0	0	0	0	0
	Chinstrap penguin	0	12	0	23	0
	Gentoo penguin	0	10	0	0	3
	Giant petrel	0	0	0	0	0
	Kelp gull	0	6	0	0	0
	Skua	0	14 (1)	0	2	0
	Snowy sheathbill	0	33 (2)	0	0	0
South Georgia	Giant petrel	4	(3)	0	0	0
	Kelp gull	6	(1)	4 (3)	0	0
	King penguin	0	0	0	0	0
	Skua	3	(3)	0	0	0
	Snowy sheathbill	5	(2)	(1)	0	0

Isolates recovered from South Georgia were identified as *C. jejuni* (18 isolates) or either *C. peloridis* (8 isolates) or *C. lari*-like bacteria (9 isolates). There were large overlaps between host species, with giant petrels and skuas carrying both *C. jejuni* and *C. lari*-like bacteria, and snowy sheathbills carrying *C. jejuni*, *C. peloridis* and *C. lari*-like bacteria (Table 2).

All but two of the 18 *C. jejuni* isolates recovered belonged to known MLST sequence types (ST-45, ST-227 and ST-883) (Table 3). Sequence types ST-45 and ST-883 were found in multiple locations and in samples from multiple host species. Sequence type ST-227 was only found in kelp gulls in Grytviken. The remaining two isolates belonged to a novel sequence type. Both isolates were from giant petrels in Stromness (Table 3).

**Table 3:** Allele numbers, sequence types (STs) and clonal complexes (CCs) of *Campylobacter jejuni* from South Georgia. New STs are shown in bold.

Location	Species	ST	*aspA*	*glnA*	*gltA*	*glyA*	*pgm*	*tkt*	*uncA*	CC
Gold Harbor	Giant petrel	45	4	7	10	4	1	7	1	ST-45
	Skua	45	4	7	10	4	1	7	1	ST-45
		883	2	17	2	3	2	1	5	ST-21
		883	2	17	2	3	2	1	5	ST-21
	Snowy sheathbill	883	2	17	2	3	2	1	5	ST-21
		883	2	17	2	3	2	1	5	ST-21
		883	2	17	2	3	2	1	5	ST-21
		883	2	17	2	3	2	1	5	ST-21
		883	2	17	2	3	2	1	5	ST-21
Grytviken	Kelp gull	45	4	7	10	4	1	7	1	ST-45
		227	2	4	5	2	2	1	5	ST-206
		227	2	4	5	2	2	1	5	ST-206
		227	2	4	5	2	2	1	5	ST-206
Stromness	Giant petrel	883	2	17	2	3	2	1	5	ST-21
		**9080**	2	1	4	28	58	25	87	ST-1332
		**9080**	2	1	4	28	58	25	87	ST-1332
	Kelp gull	45	4	7	10	4	1	7	1	ST-45
		45	4	7	10	4	1	7	1	ST-45

Of the 24 *C. lari* isolates chosen for MLST analysis, 20 could be assigned to one of 17 novel sequence types (Table 4). Of the remaining four, the *tkt* locus could not be amplified and thus no sequence type assigned.

**Table 4:** Allele numbers and sequence types (STs) of 24 *Campylobacter lari* isolates from the Antarcitc Peninsula in 2014. New STs are shown in bold.

Location	Species	ST	*adk*	*atpA*	*glnA*	*glyA*	*pgi*	*pgm*	*tkt*
Base Gabriel González Videla	Gentoo penguin	**152**	92	80	66	63	122	83	61
	Skua	**145**	67	58	85	61	78	76	57
	Snowy sheathbill	**143**	56	62	52	63	62	54	56
		**144**	96	57	1	2	58	63	52
		**149**	98	57	1	1	56	79	59
		**150**	95	80	65	63	81	81	93
		**151**	99	81	86	63	79	83	60
		–	93	78	66	63	80	80	–
		**150**	95	80	65	63	81	81	93
Kopaitik Island	Gentoo penguin	**142**	95	80	67	63	123	83	93
	Skua	**153**	62	78	1	2	1	1	32
	Snowy sheathbill	**139**	92	78	64	59	77	71	53
		–	93	78	66	63	80	72	–
		–	93	78	66	63	80	72	–
		**140**	93	78	66	63	80	73	54
		–	94	79	65	60	80	74	–
		**141**	92	78	67	63	122	75	55
		**154**	100	57	67	63	62	82	62
		**155**	101	82	68	64	82	83	63
Neko Harbor	Kelp gull	**146**	62	2	2	2	75	77	58
		**147**	2	77	2	62	58	63	33
		**147**	2	77	2	62	58	63	33
		**148**	97	57	1	2	58	78	44
	Skua	**144**	96	57	1	2	58	63	52

## Discussion

In the worst-case scenario, the introduction of novel pathogens to an ecosystem may prelude an ecological catastrophe [34]. Nevertheless, in the absence of mass mortality, the establishment of a novel pathogen may impact reproductive investment and success, which in turn may reduce the population size, disrupt the food web and increase the risk of species extinction [35, 36]. Appropriately, the threat of such introductions to the Antarctic has been recognized [7, 37]. However, whether the current measures put in place to mitigate the threat are sufficient, especially in the face of the predicted increase in human presence, has been called into question [9, 38, 39].

We isolated *Campylobacter* spp. from apparently healthy birds, as was done in previous studies [18, 40]. While the absence of overt signs of disease suggests commensal colonization rather than infection, clinical signs are rarely observed even in birds that mount an immune response to infection [41–43], and mild symptoms or opportunistic infections cannot be ruled out. Even if this is taken into account, it seems unlikely that the introduction of *Campylobacter* spp. would have a substantial adverse impact on the Antarctic ecosystem. They may, however, be used as indicators for microbial pollution, signaling areas where care must be taken lest we cause outbreaks of more virulent pathogens.

While the chosen culturing method generates the microaerobic atmosphere required for growth of most of the *Campylobacter* species previously observed in the Antarctic and sub-Antarctic, it does not generate hydrogen or formate. This excludes several species—*C. concisus*, *C. curvus*, *C. rectus*, *C. mucosalis*, *C. showae*, *C. gracilis*—that require hydrogen or formate as electron donors for microaerobic growth [10]. In addition, little is known about how different species of *Campylobacter* respond to prolonged storage in Amies medium or lysogeny broth. Barring these limitations, our findings corroborate earlier work suggesting that wild birds in the Antarctic are predominantly colonized by *C. lari* and closely related species [17–20]. Due to the limited number of studies of *C. lari* in wild birds, it is difficult to draw conclusions as to whether the isolated strains are indigenous or if the Antarctic acts as a sink, repeatedly reseeded from an outside source. Some evidence favoring the former is provided by the MLST of the 24 *C. lari* isolates yielding 17 novel sequence types, but without a clearer picture of *C. lari* host association outside of the Antarctic this remains largely speculative.

Notably, to our knowledge, this is only the second time that *C. subantarcticus* has been isolated in the wild. *C. subantarcticus*—initially described during a polyphasic taxonomic study of *C. lari*-like isolates from Bird Island, South Georgia [19]—responds well to isolation with routine protocols used in studies of other *Campylobacter* species. That it is largely absent in the literature suggests that it may be geographically restricted to the Antarctic and sub-Antarctic, restricted to the host species that occur in the region, or both. However, *Campylobacter* species other than *C. jejuni* and *C. coli* have generally received little attention and the apparent absence of *C. subantarcticus* in other regions and in non-Antarctic species may be the result of such oversight.

While we found no evidence of introduction of human-associated strains of *Campylobacter* to the Antarctic Peninsula, we did isolate such human-associated strains in South Georgia. Two of the three known sequence types recovered—ST-227 and ST-883—belong to clonal complexes frequently isolated from humans and domestic animals [44–46], but rarely from wild birds [47, 48]. The third of the three known sequence types recovered—ST-45—has frequently been isolated from humans and domestic animals [44–46], but unlike the other two is also common in wild birds [47, 49, 50].

There are several routes by which human-associated *C. jejuni* may have found its way to South Georgia. Some of the potential routes are historical and associated with the whaling era (1904–1965); alongside direct transmission from humans, these include the introduction of other known hosts for *Campylobacter*, including chickens, geese, pigeons, ducks, pigs and sheep [51]. Other potential routes may be more recent and include transmission from tourists or personnel, and yet another potential route is through transmission from remote areas by migrating birds. While the re-isolation of *C. jejuni* ST-45—the same sequence type isolated in 1998 on Bird Island, South Georgia, by Broman *et al.* [15]—may reflect persistent circulation of *C. jejuni* following a single introduction event, the presence of two additional human-associated sequence types suggests repeated introduction, but offers no further clues on the route of introduction.

In contrast to South Georgia, *C. jejuni* has never been found in the Antarctic, despite considerable monitoring effort [17, 18, 20]. The reason for this discrepancy remains unclear. Since the abandonment of the whaling stations in the 1960s, South Georgia houses no permanent residents, and personnel and tourist numbers are similar to comparable regions on the Peninsula [52, 53]. Furthermore, even though South Georgia is not encompassed by the Antarctic treaty regulations, similar management guidelines to limit the human impact are in place [52].

Thus, the presence of several human-associated MLST sequence types of *C. jejuni* in South Georgia is worrying because we do not know how they found their way there. At the same time, it is encouraging that we did not find *C. jejuni* south of the 60°S latitude—within the Antarctic Treaty Area and the pristine Antarctic—which suggests that current measures to reduce the risk of pathogen introduction may be paying off.

## Supporting information

S1 Fig Species identification of *Campylobacter* strains based on partial (1,313 bp) 16S rRNA gene sequences. (PDF)

S2 Fig Species identification of *Campylobacter* strains based on *atpA* gene sequences. Reference sequences are indicated by species names. Bootstrap values shown at nodes represent support in >95% (black), >85% (grey) and >75% (white) of 1,000 replicates, respectively.
(EPS)

S1 Table Inferred *Campylobacter* species, host species, year, region, location, sample type and method of *Campylobacter* species determination for all samples. (HTML)
