# Supplementary figures and images for: Characterization of Campylobacter spp. isolated from wild birds in the Antarctic and Sub-Antarctic
Source: PLoS One. 2018 Nov 9;13(11):e0206502. doi: 10.1371/journal.pone.0206502 (PMC6226163; doi:10.1371/journal.pone.0206502)

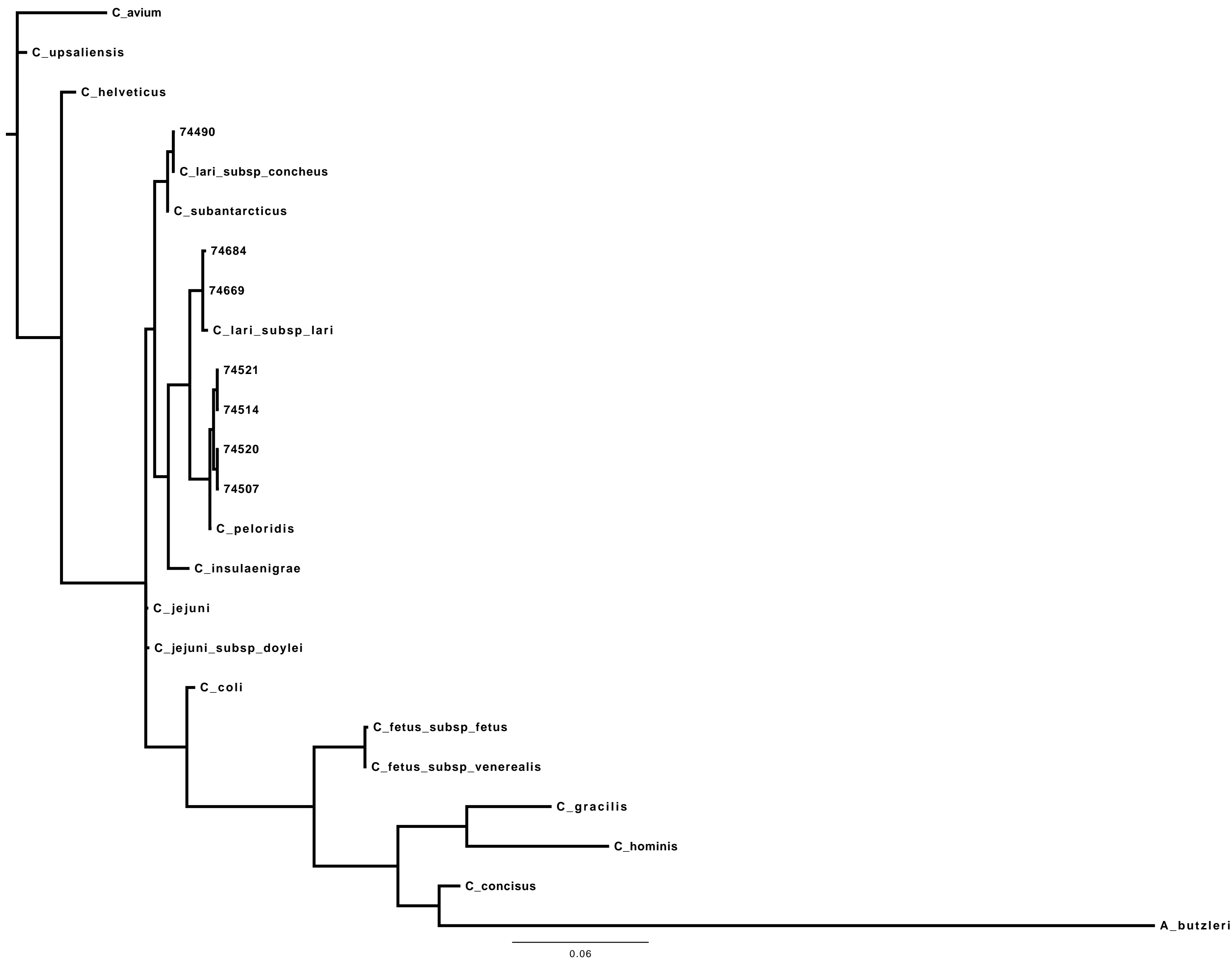

Supplement: S1 Fig — (PDF) [file pone.0206502.s001.pdf]
