# Supplementary material for: Characterization of Campylobacter spp. isolated from wild birds in the Antarctic and Sub-Antarctic
Source: PLoS One. 2018 Nov 9;13(11):e0206502. doi: 10.1371/journal.pone.0206502 (PMC6226163; doi:10.1371/journal.pone.0206502)
Supplement: S1 Table — (HTML) [file pone.0206502.s003.html]

Characterization of Campylobacter spp. isolated from wild birds in the Antarctic and Sub-Antarctic: S3 Table


# Characterization of *Campylobacter* spp. isolated from wild birds in the Antarctic and Sub-Antarctic: S3 Table

#### *Håkan Johansson, Patrik Ellström, Karin Artursson, Charlotte Berg, Jonas Bonnedahl, Ingrid Hansson, Jorge Hernandez, Juana Lopez-Martín, Gonzalo Medina-Vogel, Lucila Moreno, Björn Olsen, Eva Olsson Engvall, Hanna Skarin, Karin Troell, Jonas Waldenström, Joakim Ågren, Daniel González-Acuña*

S3 Table. Inferred *Campylobacter* species, host species, year, region, location, sample type and method of *Campylobacter* species determination for all samples.

| Sample ID | Species | Host species | Year | Region | Location | Sample type | Species determination |
| --- | --- | --- | --- | --- | --- | --- | --- |
| 0703 | *C. volucris* | Gentoo penguin | 2014 | Antarctic Peninsula | Cape Legoupil | Fecal | *atpA* sequencing |
| 0816 | *C. volucris* | Gentoo penguin | 2014 | Antarctic Peninsula | Kopaitik Island | Fecal | *atpA* sequencing |
| 1106 | *C. volucris* | Gentoo penguin | 2014 | Antarctic Peninsula | Ardley Island | Fecal | *atpA* sequencing |
| 0908 | *C. subantarcticus* | Brown skua | 2014 | Antarctic Peninsula | Base Gabriel González Videla | Cloacal swab | *atpA* sequencing |
| 0909 | *C. subantarcticus* | Brown skua | 2014 | Antarctic Peninsula | Base Gabriel González Videla | Cloacal swab | *atpA* sequencing |
| 1328 | *C. subantarcticus* | Chinstrap penguin | 2015 | Antarctic Peninsula | Cape Shirreff | Cloacal swab | *atpA* sequencing |
| 1594 | *C. subantarcticus* | Chinstrap penguin | 2015 | Antarctic Peninsula | Kopaitik Island | Cloacal swab | *atpA* sequencing |
| 1775 | *C. subantarcticus* | Chinstrap penguin | 2015 | Antarctic Peninsula | Kopaitik Island | Cloacal swab | *atpA* sequencing |
| 1792 | *C. subantarcticus* | Chinstrap penguin | 2015 | Antarctic Peninsula | Kopaitik Island | Cloacal swab | *atpA* sequencing |
| 1793 | *C. subantarcticus* | Chinstrap penguin | 2015 | Antarctic Peninsula | Kopaitik Island | Cloacal swab | *atpA* sequencing |
| 1818 | *C. subantarcticus* | Chinstrap penguin | 2015 | Antarctic Peninsula | Kopaitik Island | Cloacal swab | *atpA* sequencing |
| 1822 | *C. subantarcticus* | Chinstrap penguin | 2015 | Antarctic Peninsula | Kopaitik Island | Cloacal swab | *atpA* sequencing |
| 1823 | *C. subantarcticus* | Chinstrap penguin | 2015 | Antarctic Peninsula | Kopaitik Island | Cloacal swab | *atpA* sequencing |
| 1844 | *C. subantarcticus* | Chinstrap penguin | 2015 | Antarctic Peninsula | Kopaitik Island | Fecal | *atpA* sequencing |
| 1855 | *C. subantarcticus* | Chinstrap penguin | 2015 | Antarctic Peninsula | Kopaitik Island | Fecal | *atpA* sequencing |
| 1861 | *C. subantarcticus* | Chinstrap penguin | 2015 | Antarctic Peninsula | Kopaitik Island | Fecal | *atpA* sequencing |
| 1864 | *C. subantarcticus* | Chinstrap penguin | 2015 | Antarctic Peninsula | Kopaitik Island | Fecal | *atpA* sequencing |
| 1872 | *C. subantarcticus* | Chinstrap penguin | 2015 | Antarctic Peninsula | Kopaitik Island | Fecal | *atpA* sequencing |
| 1880 | *C. subantarcticus* | Chinstrap penguin | 2015 | Antarctic Peninsula | Kopaitik Island | Fecal | *atpA* sequencing |
| 1881 | *C. subantarcticus* | Chinstrap penguin | 2015 | Antarctic Peninsula | Kopaitik Island | Fecal | *atpA* sequencing |
| 1884 | *C. subantarcticus* | Chinstrap penguin | 2015 | Antarctic Peninsula | Kopaitik Island | Fecal | *atpA* sequencing |
| 1888 | *C. subantarcticus* | Chinstrap penguin | 2015 | Antarctic Peninsula | Kopaitik Island | Fecal | *atpA* sequencing |
| 1905 | *C. subantarcticus* | Chinstrap penguin | 2015 | Antarctic Peninsula | Kopaitik Island | Fecal | *atpA* sequencing |
| 1906 | *C. subantarcticus* | Chinstrap penguin | 2015 | Antarctic Peninsula | Kopaitik Island | Fecal | *atpA* sequencing |
| 1921 | *C. subantarcticus* | Chinstrap penguin | 2015 | Antarctic Peninsula | Kopaitik Island | Fecal | *atpA* sequencing |
| 1931 | *C. subantarcticus* | Chinstrap penguin | 2015 | Antarctic Peninsula | Kopaitik Island | Fecal | *atpA* sequencing |
| 1939 | *C. subantarcticus* | Chinstrap penguin | 2015 | Antarctic Peninsula | Kopaitik Island | Fecal | *atpA* sequencing |
| 1947 | *C. subantarcticus* | Chinstrap penguin | 2015 | Antarctic Peninsula | Kopaitik Island | Fecal | *atpA* sequencing |
| 0115 | *C. peloridis* | Kelp gull | 2012 | South Georgia | Stromness | Fecal | Phenotypic tests, PCR and MALDI-TOF |
| 0173 | *C. peloridis* | Kelp gull | 2012 | South Georgia | Grytviken | Fecal | Phenotypic tests, PCR and MALDI-TOF |
| 0174 | *C. peloridis* | Kelp gull | 2012 | South Georgia | Grytviken | Fecal | Phenotypic tests, PCR and MALDI-TOF |
| 0180 | *C. peloridis* | Kelp gull | 2012 | South Georgia | Grytviken | Fecal | Whole-genome sequencing |
| 0187 | *C. peloridis* | Kelp gull | 2012 | South Georgia | Grytviken | Fecal | Whole-genome sequencing |
| 0193 | *C. peloridis* | Kelp gull | 2012 | South Georgia | Grytviken | Fecal | Whole-genome sequencing |
| 0194 | *C. peloridis* | Kelp gull | 2012 | South Georgia | Grytviken | Fecal | Whole-genome sequencing |
| 0225 | *C. peloridis* | Snowy sheathbill | 2012 | South Georgia | Gold Harbor | Fecal | Phenotypic tests, PCR and MALDI-TOF |
| 0321 | *C. lari*-like | Kelp gull | 2012 | Antarctic Peninsula | Deception Island | Fecal | Phenotypic tests, PCR and MALDI-TOF |
| 0143 | *C. lari* | Brown skua | 2012 | South Georgia | Stromness | Fecal | Phenotypic tests, PCR and MALDI-TOF |
| 0144 | *C. lari* | Brown skua | 2012 | South Georgia | Stromness | Fecal | Phenotypic tests, PCR and MALDI-TOF |
| 0168 | *C. lari* | Kelp gull | 2012 | South Georgia | Grytviken | Fecal | Phenotypic tests, PCR and MALDI-TOF |
| 0202 | *C. lari* | Giant petrel | 2012 | South Georgia | Gold Harbor | Fecal | Phenotypic tests, PCR and MALDI-TOF |
| 0203 | *C. lari* | Giant petrel | 2012 | South Georgia | Gold Harbor | Fecal | Phenotypic tests, PCR and MALDI-TOF |
| 0205 | *C. lari* | Giant petrel | 2012 | South Georgia | Gold Harbor | Fecal | Phenotypic tests, PCR and MALDI-TOF |
| 0227 | *C. lari* | Snowy sheathbill | 2012 | South Georgia | Gold Harbor | Fecal | Phenotypic tests, PCR and MALDI-TOF |
| 0230 | *C. lari* | Snowy sheathbill | 2012 | South Georgia | Gold Harbor | Fecal | Phenotypic tests, PCR and MALDI-TOF |
| 0234 | *C. lari* | Brown skua | 2012 | South Georgia | Gold Harbor | Fecal | Phenotypic tests, PCR and MALDI-TOF |
| 0272 | *C. lari* | Brown skua | 2012 | Antarctic Peninsula | Yankee Harbor | Fecal | Phenotypic tests, PCR and MALDI-TOF |
| 0342 | *C. lari* | Snowy sheathbill | 2012 | Antarctic Peninsula | Danco Harbor | Fecal | Whole-genome sequencing |
| 0343 | *C. lari* | Snowy sheathbill | 2012 | Antarctic Peninsula | Danco Harbor | Fecal | Phenotypic tests, PCR and MALDI-TOF |
| 0344 | *C. lari* | Snowy sheathbill | 2012 | Antarctic Peninsula | Danco Harbor | Fecal | Phenotypic tests, PCR and MALDI-TOF |
| 0357 | *C. lari* | Snowy sheathbill | 2012 | Antarctic Peninsula | Orne Harbor | Fecal | Whole-genome sequencing |
| 0364 | *C. lari* | Snowy sheathbill | 2014 | Antarctic Peninsula | Kopaitik Island | Fecal | *atpA* sequencing |
| 0365 | *C. lari* | Snowy sheathbill | 2014 | Antarctic Peninsula | Kopaitik Island | Fecal | *atpA* sequencing |
| 0367 | *C. lari* | Snowy sheathbill | 2014 | Antarctic Peninsula | Kopaitik Island | Fecal | *atpA* sequencing |
| 0371 | *C. lari* | Snowy sheathbill | 2014 | Antarctic Peninsula | Kopaitik Island | Fecal | *atpA* sequencing |
| 0373 | *C. lari* | Snowy sheathbill | 2014 | Antarctic Peninsula | Kopaitik Island | Fecal | *atpA* sequencing |
| 0374 | *C. lari* | Snowy sheathbill | 2014 | Antarctic Peninsula | Kopaitik Island | Fecal | *atpA* sequencing |
| 0375 | *C. lari* | Snowy sheathbill | 2014 | Antarctic Peninsula | Kopaitik Island | Fecal | *atpA* sequencing |
| 0378 | *C. lari* | Snowy sheathbill | 2014 | Antarctic Peninsula | Cape Legoupil | Fecal | *atpA* sequencing |
| 0379 | *C. lari* | Snowy sheathbill | 2014 | Antarctic Peninsula | Cape Legoupil | Fecal | *atpA* sequencing |
| 0382 | *C. lari* | Snowy sheathbill | 2014 | Antarctic Peninsula | Cape Legoupil | Fecal | *atpA* sequencing |
| 0383 | *C. lari* | Snowy sheathbill | 2014 | Antarctic Peninsula | Cape Legoupil | Fecal | *atpA* sequencing |
| 0385 | *C. lari* | Snowy sheathbill | 2014 | Antarctic Peninsula | Cape Legoupil | Fecal | *atpA* sequencing |
| 0386 | *C. lari* | Snowy sheathbill | 2014 | Antarctic Peninsula | Cape Legoupil | Fecal | *atpA* sequencing |
| 0387 | *C. lari* | Snowy sheathbill | 2014 | Antarctic Peninsula | Cape Legoupil | Fecal | *atpA* sequencing |
| 0388 | *C. lari* | Snowy sheathbill | 2014 | Antarctic Peninsula | Cape Legoupil | Fecal | *atpA* sequencing |
| 0391 | *C. lari* | Gentoo penguin | 2014 | Antarctic Peninsula | Kopaitik Island | Cloacal swab | *atpA* sequencing |
| 0632 | *C. lari* | Snowy sheathbill | 2014 | Antarctic Peninsula | Cape Legoupil | Fecal | *atpA* sequencing |
| 0645 | *C. lari* | Gentoo penguin | 2014 | Antarctic Peninsula | Cape Legoupil | Fecal | *atpA* sequencing |
| 0692 | *C. lari* | Gentoo penguin | 2014 | Antarctic Peninsula | Cape Legoupil | Fecal | *atpA* sequencing |
| 0697 | *C. lari* | Gentoo penguin | 2014 | Antarctic Peninsula | Cape Legoupil | Fecal | *atpA* sequencing |
| 0715 | *C. lari* | Gentoo penguin | 2014 | Antarctic Peninsula | Cape Legoupil | Fecal | *atpA* sequencing |
| 0748 | *C. lari* | Gentoo penguin | 2014 | Antarctic Peninsula | Cape Legoupil | Fecal | *atpA* sequencing |
| 0895 | *C. lari* | Brown skua | 2014 | Antarctic Peninsula | Neko Harbor | Fecal | *atpA* sequencing |
| 0896 | *C. lari* | South polar skua | 2014 | Antarctic Peninsula | Base Gabriel González Videla | Cloacal swab | *atpA* sequencing |
| 0899 | *C. lari* | Snowy sheathbill | 2014 | Antarctic Peninsula | Base Gabriel González Videla | Cloacal swab | *atpA* sequencing |
| 0901 | *C. lari* | Brown skua | 2014 | Antarctic Peninsula | Neko Harbor | Cloacal swab | *atpA* sequencing |
| 0902 | *C. lari* | Brown skua | 2014 | Antarctic Peninsula | Neko Harbor | Cloacal swab | *atpA* sequencing |
| 0903 | *C. lari* | Brown skua | 2014 | Antarctic Peninsula | Base Gabriel González Videla | Cloacal swab | *atpA* sequencing |
| 0904 | *C. lari* | Brown skua | 2014 | Antarctic Peninsula | Base Gabriel González Videla | Cloacal swab | *atpA* sequencing |
| 0905 | *C. lari* | Brown skua | 2014 | Antarctic Peninsula | Base Gabriel González Videla | Cloacal swab | *atpA* sequencing |
| 0906 | *C. lari* | Brown skua | 2014 | Antarctic Peninsula | Base Gabriel González Videla | Cloacal swab | *atpA* sequencing |
| 0907 | *C. lari* | Brown skua | 2014 | Antarctic Peninsula | Base Gabriel González Videla | Cloacal swab | *atpA* sequencing |
| 0915 | *C. lari* | Gentoo penguin | 2014 | Antarctic Peninsula | Base Gabriel González Videla | Fecal | *atpA* sequencing |
| 0935 | *C. lari* | Gentoo penguin | 2014 | Antarctic Peninsula | Base Gabriel González Videla | Fecal | *atpA* sequencing |
| 0977 | *C. lari* | Kelp gull | 2014 | Antarctic Peninsula | Neko Harbor | Fecal | *atpA* sequencing |
| 0982 | *C. lari* | Kelp gull | 2014 | Antarctic Peninsula | Neko Harbor | Fecal | *atpA* sequencing |
| 0984 | *C. lari* | Kelp gull | 2014 | Antarctic Peninsula | Neko Harbor | Fecal | *atpA* sequencing |
| 0985 | *C. lari* | Kelp gull | 2014 | Antarctic Peninsula | Neko Harbor | Fecal | *atpA* sequencing |
| 0986 | *C. lari* | Kelp gull | 2014 | Antarctic Peninsula | Neko Harbor | Fecal | *atpA* sequencing |
| 0988 | *C. lari* | Kelp gull | 2014 | Antarctic Peninsula | Neko Harbor | Fecal | *atpA* sequencing |
| 1020 | *C. lari* | Snowy sheathbill | 2014 | Antarctic Peninsula | Base Gabriel González Videla | Fecal | *atpA* sequencing |
| 1022 | *C. lari* | Snowy sheathbill | 2014 | Antarctic Peninsula | Base Gabriel González Videla | Fecal | *atpA* sequencing |
| 1023 | *C. lari* | Snowy sheathbill | 2014 | Antarctic Peninsula | Base Gabriel González Videla | Fecal | *atpA* sequencing |
| 1024 | *C. lari* | Snowy sheathbill | 2014 | Antarctic Peninsula | Base Gabriel González Videla | Fecal | *atpA* sequencing |
| 1027 | *C. lari* | Snowy sheathbill | 2014 | Antarctic Peninsula | Base Gabriel González Videla | Fecal | *atpA* sequencing |
| 1028 | *C. lari* | Snowy sheathbill | 2014 | Antarctic Peninsula | Base Gabriel González Videla | Fecal | *atpA* sequencing |
| 1030 | *C. lari* | Snowy sheathbill | 2014 | Antarctic Peninsula | Base Gabriel González Videla | Fecal | *atpA* sequencing |
| 1032 | *C. lari* | Snowy sheathbill | 2014 | Antarctic Peninsula | Base Gabriel González Videla | Fecal | *atpA* sequencing |
| 1033 | *C. lari* | Snowy sheathbill | 2014 | Antarctic Peninsula | Base Gabriel González Videla | Fecal | *atpA* sequencing |
| 1034 | *C. lari* | Snowy sheathbill | 2014 | Antarctic Peninsula | Base Gabriel González Videla | Fecal | *atpA* sequencing |
| 1036 | *C. lari* | Gentoo penguin | 2014 | Antarctic Peninsula | Base Gabriel González Videla | Fecal | *atpA* sequencing |
| 1042 | *C. lari* | Gentoo penguin | 2014 | Antarctic Peninsula | Base Gabriel González Videla | Fecal | *atpA* sequencing |
| 1241 | *C. lari* | Brown skua | 2014 | Antarctic Peninsula | Cape Legoupil | Fecal | *atpA* sequencing |
| 1242 | *C. lari* | Snowy sheathbill | 2014 | Antarctic Peninsula | Cape Legoupil | Fecal | *atpA* sequencing |
| 1244 | *C. lari* | Snowy sheathbill | 2014 | Antarctic Peninsula | Cape Legoupil | Fecal | *atpA* sequencing |
| 1254 | *C. lari* | Snowy sheathbill | 2014 | Antarctic Peninsula | Cape Legoupil | Fecal | *atpA* sequencing |
| 1255 | *C. lari* | Snowy sheathbill | 2014 | Antarctic Peninsula | Cape Legoupil | Fecal | *atpA* sequencing |
| 1378 | *C. lari* | Chinstrap penguin | 2015 | Antarctic Peninsula | Cape Shirreff | Cloacal swab | *atpA* sequencing |
| 1841 | *C. lari* | Chinstrap penguin | 2015 | Antarctic Peninsula | Kopaitik Island | Fecal | *atpA* sequencing |
| 1860 | *C. lari* | Chinstrap penguin | 2015 | Antarctic Peninsula | Kopaitik Island | Fecal | *atpA* sequencing |
| 1873 | *C. lari* | Chinstrap penguin | 2015 | Antarctic Peninsula | Kopaitik Island | Fecal | *atpA* sequencing |
| 1874 | *C. lari* | Chinstrap penguin | 2015 | Antarctic Peninsula | Kopaitik Island | Fecal | *atpA* sequencing |
| 1875 | *C. lari* | Chinstrap penguin | 2015 | Antarctic Peninsula | Kopaitik Island | Fecal | *atpA* sequencing |
| 1892 | *C. lari* | Chinstrap penguin | 2015 | Antarctic Peninsula | Kopaitik Island | Fecal | *atpA* sequencing |
| 1893 | *C. lari* | Chinstrap penguin | 2015 | Antarctic Peninsula | Kopaitik Island | Fecal | *atpA* sequencing |
| 1909 | *C. lari* | Chinstrap penguin | 2015 | Antarctic Peninsula | Kopaitik Island | Fecal | *atpA* sequencing |
| 1917 | *C. lari* | Chinstrap penguin | 2015 | Antarctic Peninsula | Kopaitik Island | Fecal | *atpA* sequencing |
| 1970 | *C. lari* | Chinstrap penguin | 2015 | Antarctic Peninsula | Narębski Point | Cloacal swab | *atpA* sequencing |
| 2032 | *C. lari* | Chinstrap penguin | 2015 | Antarctic Peninsula | Narębski Point | Cloacal swab | *atpA* sequencing |
| 2247 | *C. lari* | Skua | 2016 | Antarctic Peninsula | Ardley Island | Cloacal swab | *atpA* sequencing |
| 2248 | *C. lari* | Skua | 2016 | Antarctic Peninsula | Ardley Island | Cloacal swab | *atpA* sequencing |
| 2250 | *C. lari* | Skua | 2016 | Antarctic Peninsula | Ardley Island | Cloacal swab | *atpA* sequencing |
| 2252 | *C. lari* | Skua | 2016 | Antarctic Peninsula | Ardley Island | Cloacal swab | *atpA* sequencing |
| 0097 | *C. jejuni* | Kelp gull | 2012 | South Georgia | Stromness | Fecal | *atpA* sequencing |
| 0121 | *C. jejuni* | Kelp gull | 2012 | South Georgia | Stromness | Fecal | *atpA* sequencing |
| 0125 | *C. jejuni* | Giant petrel | 2012 | South Georgia | Stromness | Fecal | *atpA* sequencing |
| 0128 | *C. jejuni* | Giant petrel | 2012 | South Georgia | Stromness | Fecal | *atpA* sequencing |
| 0137 | *C. jejuni* | Giant petrel | 2012 | South Georgia | Stromness | Fecal | *atpA* sequencing |
| 0169 | *C. jejuni* | Kelp gull | 2012 | South Georgia | Grytviken | Fecal | *atpA* sequencing |
| 0176 | *C. jejuni* | Kelp gull | 2012 | South Georgia | Grytviken | Fecal | *atpA* sequencing |
| 0181 | *C. jejuni* | Kelp gull | 2012 | South Georgia | Grytviken | Fecal | *atpA* sequencing |
| 0198 | *C. jejuni* | Kelp gull | 2012 | South Georgia | Grytviken | Fecal | *atpA* sequencing |
| 0215 | *C. jejuni* | Giant petrel | 2012 | South Georgia | Gold Harbor | Fecal | *atpA* sequencing |
| 0222 | *C. jejuni* | Snowy sheathbill | 2012 | South Georgia | Gold Harbor | Fecal | *atpA* sequencing |
| 0223 | *C. jejuni* | Snowy sheathbill | 2012 | South Georgia | Gold Harbor | Fecal | *atpA* sequencing |
| 0224 | *C. jejuni* | Snowy sheathbill | 2012 | South Georgia | Gold Harbor | Fecal | *atpA* sequencing |
| 0226 | *C. jejuni* | Snowy sheathbill | 2012 | South Georgia | Gold Harbor | Fecal | *atpA* sequencing |
| 0228 | *C. jejuni* | Snowy sheathbill | 2012 | South Georgia | Gold Harbor | Fecal | *atpA* sequencing |
| 0235 | *C. jejuni* | Brown skua | 2012 | South Georgia | Gold Harbor | Fecal | *atpA* sequencing |
| 0237 | *C. jejuni* | Brown skua | 2012 | South Georgia | Gold Harbor | Fecal | *atpA* sequencing |
| 0238 | *C. jejuni* | Brown skua | 2012 | South Georgia | Gold Harbor | Fecal | *atpA* sequencing |
